# Genetic Polymorphism of miR-196a-2 is Associated with Bone Mineral Density (BMD)

**DOI:** 10.3390/ijms18122529

**Published:** 2017-11-25

**Authors:** Irma Karabegović, Silvana Maas, Carolina Medina-Gomez, Maša Zrimšek, Sjur Reppe, Kaare M. Gautvik, André G. Uitterlinden, Fernando Rivadeneira, Mohsen Ghanbari

**Affiliations:** 1 Department of Epidemiology, Erasmus University Medical Center, ’s-Gravendijkwal 230, 3015 CE Rotterdam, the Netherlands; i.karabegovic@erasmusmc.nl (I.K.); s.maas@erasmusmc.nl (S.M.); m.medinagomez@erasmusmc.nl (C.M-G.); a.g.uitterlinden@erasmusmc.nl (A.U.); f.rivadeneira@erasmusmc.nl (F.R.); 2 Department of Internal Medicine, Erasmus University Medical Center, ’s-Gravendijkwal 230, 3015 CE Rotterdam, the Netherlands; m.zrimsek@erasmusmc.nl; 3 Department of Medical Biochemistry, Oslo University Hospital, Ullevaal, 0450 Oslo, Norway; sjur.reppe@medisin.uio.no; 4 Unger-Vetlesen Institute, Oslo Diakonale Hospital, 0456 Oslo, Norway; kaare.gautvik@lds.no; 5 Department of Molecular Medicine, University of Oslo, 0372 Oslo, Norway; 6 Department of Genetics, School of Medicine, Mashhad University of Medical Sciences, 91388-13944 Mashhad, Iran

**Keywords:** miRNA polymorphism, bone mineral density, osteoporosis, genetic variation, GWAS

## Abstract

MicroRNAs (miRNAs) are small non-coding RNA molecules that post-transcriptionally regulate the translation of messenger RNAs. Given the crucial role of miRNAs in gene expression, genetic variants within miRNA-related sequences may affect miRNA function and contribute to disease risk. Osteoporosis is characterized by reduced bone mass, and bone mineral density (BMD) is a major diagnostic proxy to assess osteoporosis risk. Here, we aimed to identify miRNAs that are involved in BMD using data from recent genome-wide association studies (GWAS) on femoral neck, lumbar spine and forearm BMD. Of 242 miRNA-variants available in the GWAS data, we found rs11614913:C > T in the precursor *miR-196a-2* to be significantly associated with femoral neck-BMD (*p*-value = 9.9 × 10^−7^, β = −0.038) and lumbar spine-BMD (*p*-value = 3.2 × 10^−11^, β = −0.061). Furthermore, our sensitivity analyses using the Rotterdam study data showed a sex-specific association of rs11614913 with BMD only in women. Subsequently, we highlighted a number of *miR-196a-2* target genes, expressed in bone and associated with BMD, that may mediate the miRNA function in BMD. Collectively, our results suggest that *miR-196a-2* may contribute to variations in BMD level. Further biological investigations will give more insights into the mechanisms by which *miR-196a-2* control expression of BMD-related genes.

## 1. Introduction

Osteoporosis is characterized by reduced bone mass and micro-architectural degradation of bone tissue, resulting in increased bone fragility, with a consequent increase in fracture susceptibility [1]. This is a common disease affecting one in three women and one in five men worldwide [2]. Incidence and development of osteoporosis increases exponentially with age [3]. The disease is diagnosed by common imaging modalities, and therefore, might be modifiable to prevent fractures [3,4]. A major diagnostic proxy to assess osteoporosis risk in the clinical field is bone mineral density (BMD) measurements, especially in skeletal sites where osteoporotic fractures occur more frequently (i.e., lumbar spine, hip and forearm) [5]. Genetic studies have estimated that 50–85% of the variance in BMD can be attributed to genetic factors [6]. A number of protein-coding genes as well as non-coding genes have been posited to contribute to osteoporosis or decreased BMD [7,8,9,10]. Functional genetics have also demonstrated eight genes that could explain up to 40% of BMD variation in postmenopausal osteoporosis and involve risk of fracture [11,12].

MicroRNAs (miRNAs) are small non-coding RNAs, approximately ~22 nucleotides long, which post-transcriptionally regulate gene expression. Together, they are estimated to regulate more than half of the genes in our genome [13]. miRNAs’ mode of action involves imperfect matching of the “seed region” (nucleotides 2–8 from the 5′ end of mature miRNA sequence) with a partially complementary sequence located at the 3′ UTR of target mRNA, resulting in translational inhibition and/or mRNA degradation [14]. It has been shown that genetic variants in miRNAs contribute to disease risk [14,15,16,17]. Polymorphisms in miRNA genes are presumed to alter miRNA biogenesis and consequently change the expression of the miRNA target genes [14,15]. This altered gene expression might result in phenotypic variation [18]. There are strong indications that miRNAs influence BMD levels by regulating several genes involved in bone-related pathways [19]. For example, *miR-146a* has been shown to regulate *TRAF6* and *IRAK1* genes involved in apoptosis [20]. In osteoclasts, these genes mediate IL-1β-induced activation of NF-κB signaling, which in turn promotes osteoclast activity and survival [21,22]. Furthermore, previous candidate gene studies have shown that genetic variants within miRNA genes (e.g., *miR-146*, *miR-125a*, *miR-27a*, *miR-433*) are associated with osteoporosis and bone cell activity, possibly through altering the miRNA expression levels or function [9,23,24,25,26].

In the present study, we hypothesized that genetic variants in miRNAs affect miRNA-mediated regulation of genes involved in BMD. To test this hypothesis, we performed a genome-wide scan for miRNA variants associated with BMD using data from the recent genome-wide association studies (GWAS) on femoral neck, lumbar spine and forearm BMD [7]. We found a genetic variant in pre-miR-196a-2 significantly associated with BMD. Subsequently, we performed in silico analyses to investigate whether *miR-196a-2* and its putative target genes may contribute to BMD variation.

## 2. Results

### 2.1. A Variant in *miR-196a-2* Associates with BMD

**Figure 1:**
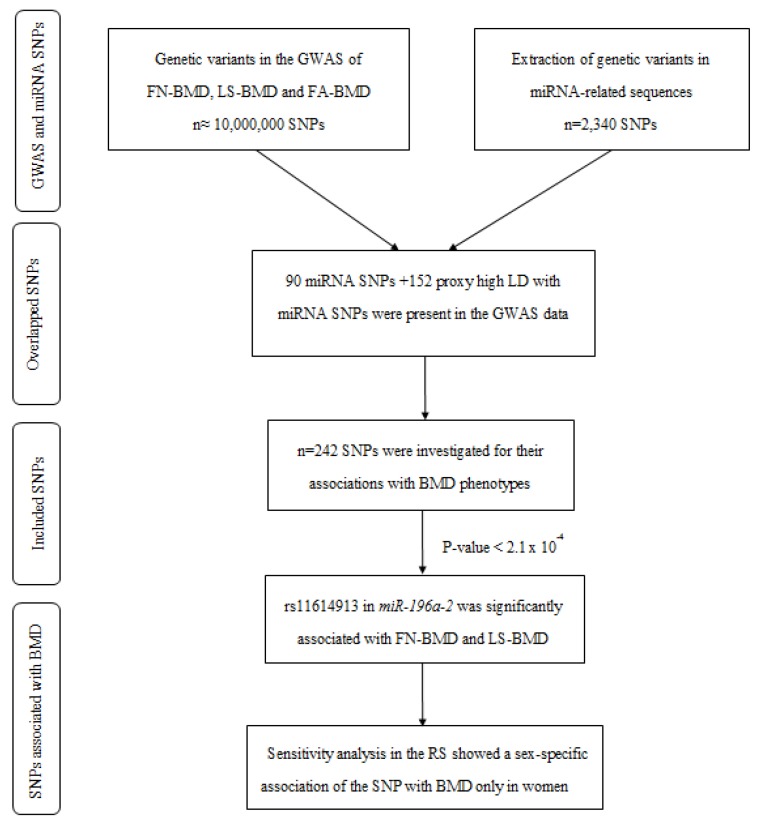
A simplified diagram of the pipeline used to identify miRNA genetic variants associated with BMD. FN-BMD: Femoral neck bone mineral density; LS-BMD: Lumbar spine bone mineral density; FA-BMD: Forearm bone mineral density; SNP: Single-nucleotide polymorphism; GWAS: Genome-wide association studies.

A total of 2340 variants in miRNA-related sequences were collected by combination of a literature review and miRNASNP database [27]. In parallel, we extracted summary statistics data from the recent GWAS meta-analysis on three BMD phenotypes, including femoral neck (FN-BMD), lumbar spine (LS-BMD) and forearm (FA-BMD), provided by Genetic Factors of Osteoporosis (GEFOS) consortium [7]. Out of 2340 miRNA variants, 90 single-nucleotide polymorphisms (SNPs) were available in the GWAS data. Using the SNAP Web tool, we extracted the proxy SNPs (*R*^2^ > 0.8 and distance < 200 kb in 1000 Genomes project) for 152 of the unavailable variants. We studied the association of these 242 miRNA SNPs with BMD phenotypes. One of the SNPs passed the Bonferroni significance threshold of 2.1 × 10^−4^ (0.05/242). This includes rs11614913:C > T in *miR-196a-2* which is significantly associated with FN-BMD (*p*-value = 9.9 × 10^−7^, β = −0.038) and LS-BMD (*p*-value = 3.2 × 10^−11^, β = −0.061). This analysis indicated that individuals carrying the rs11614913 minor allele T are more prone to have lower BMD. No significant association was identified between the miRNA variants and FA-BMD. A simplified scheme of the pipeline used for the identification of miRNA SNPs associated with the BMD phenotypes is shown in Figure 1.

### 2.2. The Potential Impact of rs11614913 on the *miR-196a-2* Structure and Function

**Figure 2:**
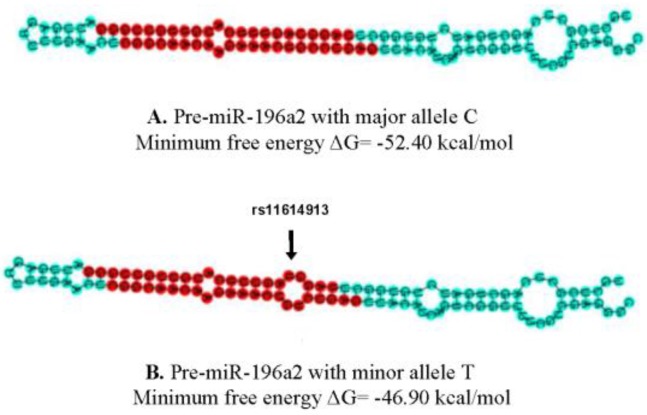
Schematic view of the predicted pre-miR-196a-2 hairpin structure containing the SNP major allele C or minor allele T. The minimum free energy (MFE) change of the thermodynamic ensemble (ΔG) is shown. The red part indicates mature sequence and the blue part shows the rest of pre-miRNA sequence.

We generated the hairpin structures of hsa-miR-196a-2 containing either the major allele C or the minor allele T at rs11614913 site using the Vienna RNAfold algorithm [28]. We observed 4.6 kcal/mol difference in the minimum free energy (MFE) of the thermodynamic predicted structure of pre-miR-196a-2 with the minor allele T compared to the wild type allele C (Figure 2). The analysis suggests that the investigated variant may affect the stability of miR-196a-2. In this line, it has been demonstrated previously that rs11614913-T decreases *miR-196a-2* expression in different cell lines [29,30].

### 2.3. Associaton of miR-196a-2 Target Genes with BMD

**Figure 3:**
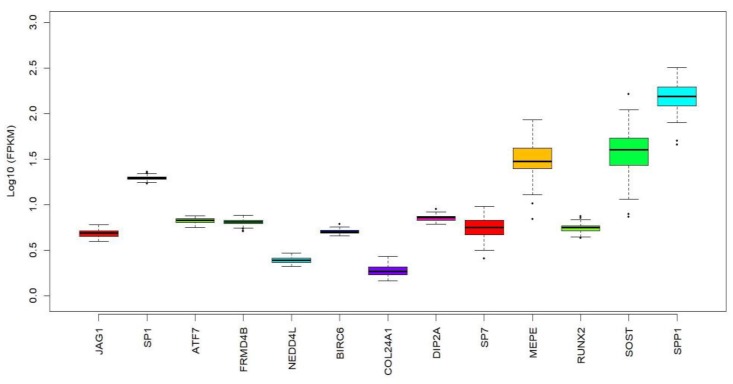
Expression of the highlighted *miR-196a-2* target genes and positive controls (*SP7*, *MEPE*, *RUNX2*, *SOST* and *SPP1*) in RNA-seq data consisting of 86 hip bone (iliac crest) biopsies. The expression data are shown in the metric Log10 FPKM (fragments per kilobase of transcript per million mapped reads).

Through leveraging the GEFOS GWAS data and using a candidate gene approach, we tested the association of genetic variants in 457 putative target genes of *miR-196a-2* with FN-BMD and LS-BMD. Table 1 shows the top ten target genes of *miR-196a-2* with the most significant association with the BMD phenotypes. Using RNA-seq gene expression data of 86 hip bone (iliac crest) biopsies, we found evidence for expression of eight out of the ten highlighted target genes of *miR-196a-2* in bone (Figure 3) [12]. Among the bone-expressed targets, *JAG1* passed the significance threshold, based on the number of variants in the tested *miR-196a-2* target genes (Table 1). This analysis may suggest that *JAG1* is more likely to mediate the downstream effect of *miR-196a-2* in relation to BMD. Moreover, a number of genes have been demonstrated experimentally (i.e., by luciferase reporter assay, Western blot or qPCR) to be regulated by *miR-196a-2*. As shown in supplementary Table S1 some of these genes are shown to be involved in either osteogenesis or bone function and may mediate the *miR-196a-2* effect on BMD. We checked the correlation of rs11614913 with expression level of its surrounding genes as shown by GTEX portal (http://www.gtexportal.org/home/) and found the association of SNP with expression of *HOXC8* and *HOXC-AS1* across different tissues.

### 2.4. Sensitivity Analyses for rs11614913 in *miR-196a-2* Using the Rotterdam Study Data

Previous studies have reported sex-specific association of genetic variants with BMD [31,32]. Furthermore, some studies have shown difference in sex response to muscoskeletal cell development, mediated by influence of steroid hormones [33,34]. In order to investigate the potential difference in association between the *miR-196a-2* variants and BMD across sexes, we performed a sensitivity analysis using the Rotterdam study (RS) data. The baseline characteristics of the RS participants are shown in Table 2. A total of 6,145 participants (3524 woman and 2621 men) from the three RS cohorts were eligible for this analysis (individuals with data available for rs11614913 and Dual X-ray Absorptiometry (DXA) imaging on FN-BMD and LS-BMD). Mixed linear regression analysis was carried out in sex-stratified data to investigate the association between rs11614913 and the BMD phenotypes (Table 3). In the basic model (adjusting for age, cohort, weight, waist to hip ratio and height) there was a significant association between rs11614913 and FN-BMD only in women (*p*-value = 0.003; β = 0.009; (95%Confidence Interval, CI) = 0.003, 0.014). The association remained significant for women in the second model (further adjusting for alcohol, smoking status and drugs used for treatment of bone diseases) (*p*-value = 0.003; β = 0.008; (95%CI) = 0.003, 0.014). We also tested the association between rs11614913 and LS-BMD and found, again, a clear significance only in women in the basic model (*p*-value = 0.023; β = 0.010; (95%CI) = 0.001, 0.019) and the second model (*p*-value = 0.026; β = 0.010; (95%CI) = 0.001, 0.018) (Table 3). Next, we further adjusted the second model for sex-hormones to see whether the miRNA variant is linked to sex-hormones (Table 3). The association in females remained significant after further adjustment for five sex-hormones (Model 3) involved in the steroidogenesis pathway. These results suggest that there is sex specificity in the association of *miR-196a-2* with BMD.

## 3. Discussion

Recent studies have shown that miRNAs are important regulators of genes linked to bone remodeling and osteoporosis development [35,36,37,38,39]. Different approaches have been used in previous studies to identify miRNAs involved in osteoporosis, including miRNA expression profiling [38,40] and candidate gene association studies [41]. In this study, we have conducted a genome-wide scan investigating the association of miRNA genetic variants with BMD using GWAS data [7]. This method represents a valuable, extended and complementary approach to previous methods used in the identification of miRNAs associated with BMD.

Our results showed that rs11614913 in the stem region of pre-miR-196a-2 is significantly associated with FN-BMD and LS-BMD. Lack of significant association between rs11614913 within pre-miR-196a-2 and forearm BMD could be attributed to the small sample size in GWAS (*n* = 8143) compared to FN-BMD (*n* = 32,735) or LS-BMD (*n* = 28,498) in the discovery cohorts [7], or differences in bone remodeling between anatomical sites. It has been shown that loaded and unloaded bone (forearm) have distinct transcriptional activities [42,43]. The location of rs11614913 in pre-miR-196a-2 is likely to affect the miRNA processing by enzyme Dicer, and subsequently alter the expression of mature *miR-196a-2* [44,45]. Polymorphisms in pre-miRNA sequences have been shown to cause either a destabilization of the interaction due to changes in the free binding energy or a change in target accessibility due to alternations in the miRNA secondary structure [19,46,47]. Our in silico analysis showed differences in the MFE between the predicted structure of pre-miR-196a-2 mutants and the wild type, suggesting the variant’s minor allele may diminish the stability of pre-miR-196a-2. In agreement with this conjecture, previous studies have established the impact of rs11614913 polymorphism (C/T) on the *miR-196a-2* expression levels [29,30,44,45,48]. Zhibin Hu et al., have reported that rs11614913 wild-type allele (C) is associated with statistically significant increase in mature *miR-196a-2* expression, while studying 23 human lung cancer tissue samples [30]. They also showed that rs11614913 could affect binding of the mature *miR-196a-2* to its candidate target mRNA [30]. Furthermore, Zhao Hauanhuan et al., observed the same trend of rs11614913*CC genotype to increase the mature *miR-196a-2* expression in different phenotypes of breast cancer [29]. Likewise, Hoffman et al., experimentally demonstrated that rs11614913 mutant allele (T) is associated with statistically significant decrease in *miR-196a-2* expression in breast cancer patients [44]. Another study by Vinci et al., presented coherent results of rs11614913*TT decreasing *miR-196a-2* expression levels in lung cancer patients [48]. In addition, Xu et al., determined that rs11614913 affects the expression of *miR-196a-2* and consequently, expression of its downstream target gene *HOXB8* [49]. They hypothesized that the variant might have an impact on *miR-196a-HOXB8-Shh* signaling pathway, and therefore, be associated with congenital heart disease susceptibility [49]. In other studies, the *miR-196a-2* polymorphism rs11614913 has been linked to various phenotypic variations, ranging from several types of cancer [30,44,45,50] to increased risk for cardiovascular disease [49,51,52,53,54]. These data strongly suggest an important functional impact of rs11614913 on *miR-196a-2* expression and function that in turn might affect the risk and/or progression of disease.

*MiR-196a* is shown to be expressed from HOX clusters loci in mammals and HOX genes in turn are shown to be targets of *miR-196a* [19,55]. The HOX genes play critical roles in limb development and skeletal patterning [56,57]. The miRNA has been also shown to play a role in brown adipogenesis of white fat progenitor cells through targeting *HOXC8* [58]. It has been proven that the miRNA regulates *HOXC8* at both mRNA and protein levels [55]. In an independent study, Kim et al., observed that adding *miR-196-a* inhibitors to osteoblast cells in culture causes a significant increase in *HOXC8* protein levels, with subsequent increased proliferation and decrease in osteogenic differentiation [59]. These data suggest upregulation of *HOXC8* in the *miR-196a-2* variant carriers, of significance for osteogenic differentiation. Accordingly, Dong-Li Zhu et al., have recently shown that *miR-196a-2* is expressed in osteoblasts and experimentally demonstrated that *FGF2*, previously identified as a susceptibility gene for osteoporosis in Caucasians [60], is a direct target of *miR-196a-2* in the Chinese population [8]. Their experiments proved that *miR-196a-2* had an influence on *FGF2* mRNA in hFOB1 cells, which is a human fetal osteoblastic cell line [8].

In addition to previously validated targets of *miR-196a-2* involved in osteogenesis, we highlighted a number of putative target genes associated with BMD with a potential to mediate the *miR-196a-2* effect in BMD. Among them, *JAG1* passed the significant threshold to be associated with BMD and is expressed in bone. The *JAG1* gene has been previously reported to be associated with increased BMD and suggested as a candidate gene for BMD regulation in diverse ethnic groups [61]. Future experimental studies are needed to explore the postulated *miR-196a-2*-mediated regulation of the gene in bone tissue or cell lines.

We performed sex-stratified analysis using the Rotterdam study data to get insight into sex specificity for BMD variation on the *miR-196a-2* polymorphism. In the sex-combined analysis, we observed significant association of rs11614913 with BMD phenotypes. However, sex-stratified analysis revealed that the association is mainly driven by women. We acknowledge that the observed association in women may have been driven by a lower number of men (our cohort contains 903 more women than men), however, sample size of 6145 should be sustainable to address sex difference. Notably, the *miR-196a-2* polymorphism rs11614913 with combination of rs3746444 in *miR-499a* have been reported previously to be involved in the multiple sclerosis severity, where the association shows only female sex specificity [62]. Multiple sclerosis and osteoporosis share a surprising number of risk factors [63,64,65] and genetics might be one of them, although the interplay of the two miRNA variants and their impacts on gene interaction should be taken in consideration when interpreting the results regarding sex specificity. Considering the sexual dimorphism of bone [31,66], these data might indicate a potential for further clinical and biological investigations regarding the role of *miR-196a-2* underlying BMD variation.

This study has some strengths and limitations that need to be considered in interpretation of the reported results. The major strength of this study is leveraging genetic data from the recent GWAS of BMD phenotypes that enabled us legitimate statistical power for detection of miRNA-related variants associated with BMD. The main limitation that needs to be addressed is lack of experimental studies in relevant tissues or cell lines. MiRNA-related SNPs might be only utilitarian if the target mRNA is expressed in the same tissue [67]. Thereby, further biological investigations warrant better insights into the mechanisms by which *miR-196a-2* control expression of genes involved in BMD.

## 4. Materials and Methods

### 4.1. Genome-Wide Association Studies on BMD Phenotypes

The summary statistics from the recent GWAS meta-analysis on FN-BMD (*n* = 32,735), LS-BMD (*n* = 28,498) and FA-BMD (*n* = 8143) provided by GEFOS consortium were extracted [7]. The GEFOS consortium is a collective effort of numerous research groups combining GWAS data, in order to identify osteoporosis susceptibility alleles that regulate BMD and fracture risk [7]. The GEFOS consortium performed meta-analysis of whole genome sequencing, whole exome sequencing and deep imputation of genotype data in order to determine low-frequency and rare variants associated with risk factors for osteoporosis. The collaboration within the GEFOS has resulted in producing files with summary statistics for approximately 10 million genetics variants (the 1000 Genomes/UK10K reference panel) in 53,236 individuals [7]. More details on datasets and participants are described in detail elsewhere [7].

### 4.2. Identification of Genetic Variants in miRNA-Encoding Sequences

A dataset of single-nucleotide polymorphisms (SNPs) in miRNA-related sequences was created by combining miRNASNP (http://www.bioguo.org/miRNASNP/) [27] and the literature review (searching in PubMed for miRNA genetic variants). Precursor miRNA sequences (pre-miRNA) undergo cleavage by enzyme Dicer, yielding to mature miRNAs [13], therefore we screened all variants located in human pre-miRNA and mature miRNA sequences. The methodology was explained in details elsewhere [68]. Variants with minor allele frequency (MAF) >0.01 were included. Variants with smaller MAF were illegible due to low imputation quality and issue of being underpowered in further studies. In total, 2340 miRNA variants were extracted. Of these, 242 variants were available in the GEFOS GWAS data and were therefore investigated further for their associations with BMD phenotypes.

### 4.3. miRNA Target Genes Associated with BMD Phenotypes

Once a miRNA variant was found to be significantly associated with BMD phenotypes, we searched for the miRNA target genes. We postulated that some of the miRNA target genes may mediate the downstream effect of miRNA in relation to BMD phenotypes. In order to identify target genes of miRNAs, putative target genes were extracted from combining TargetScan v7.1 (http://www.targetscan.org/vert_71/) and miRDB (http://mirdb.org/) database [69]. Target genes present in both databases were selected for further investigation. Any supplementary information, such as miRNA conservation between species, host genes, miRNA sequences was collected from TargetScan (v7.1). Both context score and conserved target sites were used to rank the miRNA target genes. In addition, the online database, miRTarBase (http://mirtarbase.mbc.nctu.edu.tw/) provides information on various functional experiments, such as microarrays, western blot, and reported assays performed between miRNAs and their target genes [70]. We used miRTarBase to search for functional experiment confirming the putative interaction between miRNAs of interest and their target genes. A candidate gene approach was performed by leveraging the GWAS data on BMD phenotypes [7] and to investigate the association between genetic variants in the miRNA target genes and BMD. In addition, we evaluated the expression of selected target genes in the bone tissue. Dataset used for gene expression was created out of 86 iliac biopsies [12].

### 4.4. The Variant Effect on the Pre-miRNA Structure

The secondary structure of pre-miRNA is critical for the miRNA production. The Vienna RNAfold algorithm (ViennaRNA package 2.0) was used to predict the impact of miRNA variants on the hairpin stem-loop structure of pre-miRNAs [28]. The ViennaRNA package 2.0 is available to the public domain and relies on numerous algorithms for prediction and analysis of RNA secondary structures [71]. The program calculates the shift in minimum free energy (MFE) of the thermodynamic ensemble in the hairpin structure of miRNA (wild type and mutant) [72]. The shift in MFE is likely to be related to the function, as it can result in instability of miRNA.

### 4.5. The Rotterdam Study Data

The Rotterdam study (RS) is a population-based cohort study, with main goal of identifying chronic disabling conditions of the middle aged and elderly people [73]. Participants were interviewed at home and went through an extensive set of examinations, including bone mineral densitometry, sample collections for in-depth molecular and genetic analysis [73]. The RS includes three sub-cohorts. We used the data from the baseline, second and third cohort (RS-I-4, RS-II-2, and RS-III-1). For all participants, DXA-based BMD measurements were collected for FN-BMD and LS-BMD. The RS does not include data on FA-BMD since this site is used for prediction of osteoporosis only when data is not available for FN-BMD or LS-BMD due to numerous reasons (e.g., patients either being obese, men with hyperparathyroidism or receiving androgen-deprivation therapy (ADT) for prostate cancer) [74]. Furthermore, determinants were assessed either by physical examinations, collection of blood samples, or by questionnaires. Participants were included if they had FN-BMD or LS-BMD measurements, which resulted in combination of three cohorts (RS-I-4, RS-II-2, and RS-III-1). We used multiple linear regression in sex-stratified dataset to examine the association between the candidate miRNA variant and BMD phenotypes (separately). Our analysis was adjusted for all potential confounders in three models.

## 5. Conclusions

The results of this study suggest that *miR-196a-2* polymorphism (rs11614913:C > T) is associated with reduced FN-BMD and LS-BMD. We highlighted a number of target genes that may mediate *miR-196a-2* function in influencing BMD. The identified *miR-196a-2* might have a future implication in the clinical field related to diagnosis and treatment of osteoporosis. Future biological studies will give insight into the mechanisms by which *miR-196a-2* may control expression of bone-related genes. Collectively, our study provides further understanding of the miRNA-mediated regulation of BMD.

## Figures and Tables

**Table 1:** Putative target genes of *miR-196a-2* (3p and 5p) that are associated with FN-BMD and LS-BMD. Leading SNPs within each target gene associated with BMD in GEFOS GWAS data are shown. Significantly associated genes, after Bonferroni correction for multiple testing (*p*-value <7.0 × 10^−6^), are depicted in bold.

miRNA ID	Associated Phenotype	Associated Target Genes	*p-*Value in GWAS Data	Top SNP
miR-196a-3p	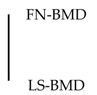	*JAG1*	1.8 × 10^−5^	rs2235811
		*MACROD2*	2.0 × 10^−6^	rs365824
		*SP1*	4.2 × 10^−5^	rs4759334
		*JAG1*	4.7 × 10^−9^	rs2235811
		*ATF7*	6.3 × 10^−5^	rs1078358
		*MACROD2*	8.1 × 10^−5^	rs6110288
miR-196a-5p	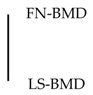	*FRMD4B*	5.6 × 10^−4^	rs1564757
		*NEDD4L*	9.6 × 10^−4^	rs533502
		*BIRC6*	1.2 × 10^−3^	rs6737916
		*COL24A1*	2.6 × 10^−3^	rs1359419
		*RSPO2*	3.1 × 10^−3^	rs446454
		*DIP2A*	3.3 × 10^−3^	rs2330593

**Table 2:** Demographic characteristics of the Rotterdam study cohorts. Values are mean (standard deviation), numbers (percentages) or median (interquartile range (IQR)); used for alcohol only. FN-BMD: Femoral neck bone mineral density; LS-BMD: Lumbar spine bone mineral density; WHR: Waist to hip ratio; Bone drugs: drugs used for treatment of bone diseases; DHEA: dehydroepiandrosterone; DHEAS: dehydroepiandrosterone sulfate.

Variables		Men	Women
FN-BMD (g/cm^2^)		0.95 (0.14)	0.87 (0.14)
LS-BMD (g/cm^2^)		1.20 (0.19)	1.08 (0.19)
Age (years)		65.71 (10.45)	66.29 (10.61)
Weight (kg)		85.55 (12.85)	73.11 (13.09)
WHR		0.95 (0.07)	0.84 (0.07)
Height (cm)		176.41 (7.01)	162.73 (6.50)
Alcohol (g/day)		9.29 (3.57–20.00)	4.29 (0.54–10.00)
DHEA (nmol/L)		11.82 (7.32)	12.31 (7.65)
DHEAS (nmol/L)		3200.18 (1757.16)	2099.17 (1337.77)
Androstenedione (nmol/L)		3.24 (1.27)	2.70 (1.29)
Testosterone (nmol/L)		17.53 (5.78)	0.90 (0.45)
Estradiol (pmol/L)		96.93 (33.82)	38.86 (33.18)
Smoking	never smoker	1125 (42.9%)	2071 (58.8%)
	former smoker	1039 (39.7%)	841 (23.9%)
	current smoker	456 (17.4%)	612 (17.4%)
Bone drugs	no	2607 (99.5%)	3400 (96.5%)
	yes	13 (0.5%)	124 (3.5%)

**Table 3:** Association between rs11614913 and BMD phenotypes in participants of the Rotterdam Study. Model 1 (M1) is adjusted for age, cohort, weight, waist to hip ratio (WHR) and height. Model 2 (M2) is adjusted for M1 + alcohol, smoking status (current, former and never smoker) and drugs used for treatment of bone diseases. Model 3 (M3) is adjusted for M2 + estradiol, testosterone, androstenedione, DHEA, and DHEAS. “Combined” was additionally adjusted for sex.

Phenotype	Model	Men			Women			Combined		
		β	95%CI	*p*-Value	β	95%CI	*p*-v	β	95%CI	*p*-Value
FN-BMD	M1	0.004	−0.003, 0.011	0.257	0.009	0.003, 0.014	0.003	0.007	0.003, 0.012	0.002
	M2	0.004	−0.003, 0.011	0.267	0.008	0.003, 0.014	0.003	0.007	0.003, 0.012	0.002
	M3	0.004	−0.004, 0.011	0.319	0.008	0.003, 0.014	0.003	0.007	0.002, 0.011	0.003
LS-BMD	M1	0.005	−0.006, 0.016	0.380	0.010	0.001, 0.019	0.023	0.009	0.002, 0.016	0.011
	M2	0.004	−0.007, 0.015	0.423	0.010	0.001, 0.018	0.026	0.009	0.002, 0.016	0.012
	M3	0.003	−0.008, 0.014	0.573	0.009	0.001, 0.018	0.038	0.008	0.001, 0.015	0.020

## Supplementary Materials

Supplementary materials can be found at www.mdpi.com/1422-0067/18/12/2529/s1.

## Abbreviations

GWAS	Genome-wide association studies
GEFOS	Genetic factors for osteoporosis
BMD SNP	Bone mineral density Single nucleotide polymorphism
FN-BMD	Femoral neck bone mineral density
LS-BMD	Lumbar spine bone mineral density
FA-BMD	Forearm bone mineral density
miRNA	microRNA
WHR	Waist to hip ratio
RS	Rotterdam Study
DXA	Dual X-ray Absorptiometry
MFE	Minimum free energy
LD	Linkage disequilibrium
DHEA	Dehydroepiandrosterone
DHEAS	Dehydroepiandrosterone sulfate
IQR	Interquartile range
