# Supplementary material for: Genetic Polymorphism of miR-196a-2 is Associated with Bone Mineral Density (BMD)
Source: Int J Mol Sci. 2017 Nov 25;18(12):2529. doi: 10.3390/ijms18122529 (PMC5751132; doi:10.3390/ijms18122529)
Supplement: Supplementary file 1 [file ijms-18-02529-s001.pdf]

Supplementary Table S1. Target genes of miR-196a that have been validated experimentally (reporter assay, western blot or qPCR). Genes which have been previously associated with either osteogenesis or bone function are depicted in bold.

Gene	Gene Name	Function
ANXA1 [1]	Annexin A1	calcium ion binding, receptor binding
HOXB8 [2–4]	Homeobox protein Hox-B8	transcription factor activity, sequence-specific DNA binding and sequence-specific DNA binding
HOXC8 [5,6]	Homeobox protein Hox-C8	transcription factor activity, sequence-specific DNA binding and sequence-specific DNA binding.
HOXB7 [7–9]	Homeobox protein Hox-B7	transcription factor activity, sequence-specific DNA binding and sequence-specific DNA binding
BACH1 [10,11]	BTB Domain and CNC Homolog 1	transcription factor activity, sequence-specific DNA binding and heme binding
HMOX1 [12–19]	Heme Oxygenase 1	protein homodimerization activity and oxidoreductase activity
CDKN1B	Cyclin Dependent Kinase Inhibitor 1B	protein complex binding and protein phosphatase binding
HMGA1	High Mobility Group AT-Hook 1	enzyme binding , ligand-dependent nuclear receptor transcription coactivator activity.
HMGA2 [20–23]	High mobility Group AT-Hook 2	enzyme binding , transcriptional activator activity, RNA polymerase II core promoter proximal region sequence-specific binding
HOXA5 [24–26]	Homeobox A5	transcription factor activity, sequence-specific DNA binding and RNA polymerase II core promoter proximal region sequence-specific DNA binding
FOXO1 [27–29]	Forkhead Box O1	transcription factor activity, sequence-specific DNA binding and chromatin binding
RDX	Radixin	poly[A] RNA binding and cytoskeletal protein binding
NFKBIA [30,31]	NFKB Inhibitor Alpha	identical protein binding and transcription factor binding
HOXA7	Homeobox A7	transcription factor activity, sequence-specific DNA binding and transcription factor binding
HOXD8	Homeobox D8	transcription factor activity, sequence-specific DNA binding and RNA polymerase II regulatory region sequence-specific DNA binding
SPRR2C	Small Proline Rich Protein 2C	pseudogene
S100A9 [32]	S100 Calcium Binding Protein A9	calcium ion binding and microtubule binding
KRT5	Keratin 5	structural molecule activity and scaffold protein binding
NTN4	Netrin 4	laminin-1 binding
LLGL1	Scribble Cell Polarity Complex Component	protein kinase binding and structural molecule activity

References

1. Suarez F, Rothhut B, Comera C, Touqui L, Marie FR, Silve C. Expression of annexin I, II, V, and VI by rat osteoblasts in primary culture: stimulation of annexin I expression by dexamethasone. J Bone Miner Res. 1993 Oct;8[10]:1201-10.
2. van den Akker E, Fromental-Ramain C, de Graaff W, Le Mouellic H, Brulet P, Chambon P, et al. Axial skeletal patterning in mice lacking all paralogous group 8 Hox genes. Development. 2001 May;128[10]:1911-21.
3. McGlinn E, Yekta S, Mansfield JH, Soutschek J, Bartel DP, Tabin CJ. In ovo application of antagomiRs indicates a role for miR-196 in patterning the chick axial skeleton through Hox gene regulation. Proc Natl Acad Sci U S A. 2009 Nov 03;106[44]:18610-5.
4. Zach F, Mueller A, Gessner A. Production and Functional Characterization of Murine Osteoclasts Differentiated from ER-Hoxb8-Immortalized Myeloid Progenitor Cells. PLoS One. 2015;10[11]:e0142211.
5. Kruger C, Kappen C. Expression of cartilage developmental genes in Hoxc8- and Hoxd4-transgenic mice. PLoS One. 2010 Feb 02;5[2]:e8978.
6. Kim YJ, Bae SW, Yu SS, Bae YC, Jung JS. miR-196a regulates proliferation and osteogenic differentiation in mesenchymal stem cells derived from human adipose tissue. J Bone Miner Res. 2009 May;24[5]:816-25.
7. Gao RT, Zhan LP, Meng C, Zhang N, Chang SM, Yao R, et al. Homeobox B7 promotes the osteogenic differentiation potential of mesenchymal stem cells by activating RUNX2 and transcript of BSP. Int J Clin Exp Med. 2015;8[7]:10459-70.
8. Candini O, Spano C, Murgia A, Grisendi G, Veronesi E, Piccinno MS, et al. Mesenchymal progenitors aging highlights a miR-196 switch targeting HOXB7 as master regulator of proliferation and osteogenesis. Stem Cells. 2015 Mar;33[3]:939-50.
9. Chen F, Greer J, Capecchi MR. Analysis of Hoxa7/Hoxb7 mutants suggests periodicity in the generation of the different sets of vertebrae. Mech Dev. 1998 Sep;77[1]:49-57.
10. Hama M, Kirino Y, Takeno M, Takase K, Miyazaki T, Yoshimi R, et al. Bach1 regulates osteoclastogenesis in a mouse model via both heme oxygenase 1-dependent and heme oxygenase 1-independent pathways. Arthritis Rheum. 2012 May;64[5]:1518-28.
11. Kanzaki H, Shinohara F, Itohiya K, Yamaguchi Y, Katsumata Y, Matsuzawa M, et al. RANKL induces Bach1 nuclear import and attenuates Nrf2-mediated antioxidant enzymes, thereby augmenting intracellular reactive oxygen species signaling and osteoclastogenesis in mice. FASEB J. 2017 Feb;31[2]:781-92.
12. Davis C, Dukes A, Drewry M, Helwa I, Johnson MH, Isales CM, et al. MicroRNA-183-5p Increases with Age in Bone-Derived Extracellular Vesicles, Suppresses Bone Marrow Stromal [Stem] Cell Proliferation, and Induces Stem Cell Senescence. Tissue Eng Part A. 2017 Apr 28.
13. Vanella L, Kim DH, Asprinio D, Peterson SJ, Barbagallo I, Vanella A, et al. HO-1 expression increases mesenchymal stem cell-derived osteoblasts but decreases adipocyte lineage. Bone. 2010 Jan;46[1]:236-43.
14. Zwerina J, Tzima S, Hayer S, Redlich K, Hoffmann O, Hanslik-Schnabel B, et al. Heme oxygenase 1 [HO-1] regulates osteoclastogenesis and bone resorption. FASEB J. 2005 Dec;19[14]:2011-3.
15. Barbagallo I, Vanella A, Peterson SJ, Kim DH, Tibullo D, Giallongo C, et al. Overexpression of heme oxygenase-1 increases human osteoblast stem cell differentiation. J Bone Miner Metab. 2010 May;28[3]:276-88.
16. Yuan TL, Chen J, Tong YL, Zhang Y, Liu YY, Wei JC, et al. Serum Heme Oxygenase-1 and BMP-7 Are Potential Biomarkers for Bone Metabolism in Patients with Rheumatoid Arthritis and Ankylosing Spondylitis. Biomed Res Int. 2016;2016:7870925.
17. Suttorp CM, Xie R, Lundvig DM, Kuijpers-Jagtman AM, Uijttenboogaart JT, Van Rheden R, et al. Orthodontic Forces Induce the Cytoprotective Enzyme Heme Oxygenase-1 in Rats. Front Physiol. 2016;7:283.

18. Kang H, Yan Y, Jia P, Yang K, Guo C, Chen H, et al. Desferrioxamine reduces ultrahigh-molecular-weight polyethylene-induced osteolysis by restraining inflammatory osteoclastogenesis via heme oxygenase-1. Cell Death Dis. 2016 Oct 27;7[10]:e2435.
19. Bak SU, Kim S, Hwang HJ, Yun JA, Kim WS, Won MH, et al. Heme oxygenase-1 [HO-1]/carbon monoxide [CO] axis suppresses RANKL-induced osteoclastic differentiation by inhibiting redox-sensitive NF-kappaB activation. BMB Rep. 2017 Feb;50[2]:103-8.
20. Kuipers A, Zhang Y, Cauley JA, Nestlerode CS, Chu Y, Bunker CH, et al. Association of a high mobility group gene [HMGA2] variant with bone mineral density. Bone. 2009 Aug;45[2]:295-300.
21. Dahlen A, Mertens F, Rydholm A, Brosjo O, Wejde J, Mandahl N, et al. Fusion, disruption, and expression of HMGA2 in bone and soft tissue chondromas. Mod Pathol. 2003 Nov;16[11]:1132-40.
22. Wei J, Li H, Wang S, Li T, Fan J, Liang X, et al. let-7 enhances osteogenesis and bone formation while repressing adipogenesis of human stromal/mesenchymal stem cells by regulating HMGA2. Stem Cells Dev. 2014 Jul 01;23[13]:1452-63.
23. Wang H, Sun Z, Wang Y, Hu Z, Zhou H, Zhang L, et al. miR-33-5p, a novel mechano-sensitive microRNA promotes osteoblast differentiation by targeting Hmga2. Sci Rep. 2016 Mar 16;6:23170.
24. Yerges LM, Klei L, Cauley JA, Roeder K, Kammerer CM, Moffett SP, et al. High-density association study of 383 candidate genes for volumetric BMD at the femoral neck and lumbar spine among older men. J Bone Miner Res. 2009 Dec;24[12]:2039-49.
25. Li N, Sun S, Wang D, Yao P, Yang X, Yan H, et al. Suppression of retinoic acid receptors may contribute to embryonic skeleton hypoplasia in maternal rats with chronic vitamin A deficiency. J Nutr Biochem. 2010 Aug;21[8]:710-6.
26. Tabaries S, Lapointe J, Besch T, Carter M, Woollard J, Tuggle CK, et al. Cdx protein interaction with Hoxa5 regulatory sequences contributes to Hoxa5 regional expression along the axial skeleton. Mol Cell Biol. 2005 Feb;25[4]:1389-401.
27. Rached MT, Kode A, Xu L, Yoshikawa Y, Paik JH, Depinho RA, et al. FoxO1 is a positive regulator of bone formation by favoring protein synthesis and resistance to oxidative stress in osteoblasts. Cell Metab. 2010 Feb 03;11[2]:147-60.
28. Sasanuma H, Nakata M, Parmila K, Nakae J, Yada T. PDK1-FoxO1 pathway in AgRP neurons of arcuate nucleus promotes bone formation via GHRH-GH-IGF1 axis. Mol Metab. 2017 May;6[5]:428-39.
29. Dixit M, Singh KB, Prakash R, Singh D. Functional block of IL-17 cytokine promotes bone healing by augmenting FOXO1 and ATF4 activity in cortical bone defect model. Osteoporos Int. 2017 Jul;28[7]:2207-20.
30. Liu YZ, Zhou Y, Zhang L, Li J, Tian Q, Zhang JG, et al. Attenuated monocyte apoptosis, a new mechanism for osteoporosis suggested by a transcriptome-wide expression study of monocytes. PLoS One. 2015;10[2]:e0116792.
31. Greenblatt MB, Park KH, Oh H, Kim JM, Shin DY, Lee JM, et al. CHMP5 controls bone turnover rates by dampening NF-kappaB activity in osteoclasts. J Exp Med. 2015 Jul 27;212[8]:1283-301.
32. Zreiqat H, Howlett CR, Gronthos S, Hume D, Geczy CL. S100A8/S100A9 and their association with cartilage and bone. J Mol Histol. 2007 Oct;38[5]:381-91.
